# Prevalence of the metabolic syndrome and determination of optimal cut-off values of waist circumference in university employees from Angola

**DOI:** 10.5830/CVJA-2013-086

**Published:** 2014-02

**Authors:** Pedro Magalhães, Daniel P Capingana, José G Mill

**Affiliations:** Department of Physiology, Faculty of Medicine, University Agostinho Neto, Luanda, Angola; Department of Physiology, Faculty of Medicine, University Agostinho Neto, Luanda, Angola; Department of Physiology, Federal University of Espírito Santo, Vitoria, Brazil

**Keywords:** metabolic syndrome, waist circumference, Africans, Angola

## Abstract

**Background:**

Estimates of the prevalence of the metabolic syndrome in Africans may be inconsistent due to lack of African-specific cut-off values of waist circumference (WC). This study determined the prevalence of the metabolic syndrome and defined optimal values of WC in Africans.

**Methods:**

This cross-sectional study collected demographic, anthropometric and clinical data of 615 Universitary employees, in Luanda, Angola. The metabolic syndrome was defined using the third report of the National Cholesterol Education Program Adult Treatment Panel (ATPIII) and the Joint Interim Statement (JIS) criteria. Receiver operating characteristics curves were constructed to assess cut-off values of WC.

**Results:**

The crude prevalence of the metabolic syndrome was higher with the JIS definition (27.8%, age-standardised 14.1%) than with the ATP III definition (17.6%, age-standardised 8.7%). Optimal cut-off values of WC were 87.5 and 80.5 cm in men and women, respectively.

**Conclusions:**

There was a high prevalence of the metabolic syndrome among our African subjects. Our data suggest different WC cut-off values for Africans in relation to other populations.

## Abstract

The metabolic syndrome is characterised by the presence of multiple metabolic risk factors for cardiovascular (CV) disease^1^ and type 2 diabetes mellitus.^2^ In clinical practice, the metabolic syndrome is diagnosed by combinations of three or more of the following five risk factors: central obesity, elevated blood pressure, glucose intolerance, hypertriglyceridaemia and low high-density lipoprotein cholesterol (HDL-C).^3^-^6^

Worldwide the prevalence of the metabolic syndrome is increasing and becoming a pandemic, and this increase has been mainly attributed to sedentary lifestyle and obesity.^7^ However, levels of prevalence may vary greatly according to cut-off points of diagnostic criteria and the ethnic group studied.^8^

In sub-Saharan Africa, the majority of countries are experiencing a rapid demographic and epidemiological transition.^9^,^10^ Available information from studies in African populations reported a prevalence of the metabolic syndrome ranging from 0% to as high as about 50% or more, depending on the population setting.^11^ These data however, are limited to some countries,^12^-^21^ since there are no available data for the majority of African countries.

Angola is a country in sub-Saharan Africa, which in the last few years has undergone significant political changes, accompanied by a rapid economic growth and increased urbanisation. These changes may imply an increasing prevalence of factors contributing to the metabolic syndrome, such as obesity, insufficient physical activity, dyslipidaemia, high blood pressure and glucose intolerance. However, the prevalence of the metabolic syndrome and which factors contribution more to its occurrence in the Angolan population remain unknown.

Despite the efforts of several organisations to regulate the algorithm for a definition of the metabolic syndrome,^3^-^5^ there is inconsistency on cut-off levels of waist circumference (WC) for defining the metabolic syndrome in several populations. The International Diabetes Federation (IDF)^5^ recommended the use of ethnic or country-specific cut-off values of WC for the majority of populations, a recommendation reinforced in the Joint Interim Statement (JIS),^7^ which tried to define different criteria for a definition of the metabolic syndrome.

These cut-off values were defined using different methods. For example, Western countries derived their cut-off values of WC from a correlation with body mass index (BMI),^4^,^22^ whereas Asian groups tried to define WC cut-off values yielded by receiver operating characteristics (ROC) curve analyses.^23^ Due to a lack of specific data from African populations, cut-off points of WC derived from the European population have been recommended,^5^,^7^ although emerging data suggest that African-specific cut-off values would be different from the European cut-off points currently recommended by the IDF.^18^,^24^,^25^ Therefore, definition of a more reliable cut-off point for WC is needed to build a consistent tool for diagnosis of the metabolic syndrome in sub-Saharan African populations.

The aim of this study was to determine the prevalence of the metabolic syndrome in a sample of Africans from Angola, using either the third report of the National Cholesterol Education Program Adult Treatment Panel (ATP III)^4^ or the JIS^7^ criteria. Additionally, this study tried to identify threshold WC levels that best predict other components of the metabolic syndrome.

## Methods

This was a cross-sectional study on cardiovascular (CV) risk factors, conducted from 2009 to 2010 in employees of a public university in Luanda, Angola. Participants aged 20 years and older (*n* = 625) visited the Department of Physiology, Faculty of Medicine of Agostinho Neto University, Luanda, Angola to be submitted to clinical and laboratorial examinations to identify cardiovascular risk.

A total of 615 subjects with complete data were included in this study. Details of the study design are described elsewhere.^26^,^27^ The study was conducted according to the tenets of the Declaration of Helsinki and participants signed an informed consent form approved by the Ethics Committee of the Faculty of Medicine, Agostinho Neto University.

Clinical examinations were performed between 08:00 and noon in temperature-controlled rooms (22–23°C) after a 2-hour fast. Participants were asked to refrain from smoking, physical exercise and caffeinated beverages for at least 12 hours before the visit. Venous blood samples were obtained from the forearm by standard techniques and processed immediately using commercially available kits (BioSystems SA, Costa Brava 30, Barcelona, Spain) for determination of levels of serum triglycerides, total cholesterol, high-density lipoprotein cholesterol (HDL-C), glucose, creatinine and uric acid.

Biochemical parameters were analysed using enzymatic methods on a spectrophotometer (BioSystems BTS-310, Barcelona, Spain). In subjects with triglyceride levels < 400 mg/dl (4.52 mmol/l), low-density lipoprotein cholesterol (LDL-C) was calculated according to Friedewald’s formula,^28^ and very low-density lipoprotein cholesterol (VLDL-C) was calculated as previously described.^4^

Diabetes was defined as a fasting glucose level ≥ 126 mg/dl (6.99 mmol/l) or the use of antidiabetic drugs.^29^ Dyslipidaemia was defined as the presence of one or more of the following: total cholesterol ≥ 200 mg/dl (5.18 mmol/l), triglycerides ≥ 150 mg/dl (1.70 mmol/l), LDL-C ≥ 160 mg/dl (4.14 mmol/l), or HDL-C < 40 mg/dl (1.04 mmol/l) (men), < 50 mg/dl (1.30 mmol/l) (women).^4^

Demographics including socio-economic level, educational data and medical history were collected using a structured questionnaire. Participants were classified as non-smokers (never and ex-smokers) and current smokers (daily and occasional smokers).

Anthropometric measures included weight, height, WC and hip circumference (HC), obtained from individuals wearing underwear and no shoes. Weight was measured to the nearest 0.1 kg using a previously calibrated mechanical scale (SECA GmbH & Co, Germany) with a maximum capacity of 220 kg.

Height was measured with a precision of 0.5 cm using a stadiometer fixed to the SECA scale. WC and HC were each measured twice using an inextensible, 1-cm-wide tape measure. The WC was measured at the end of normal expiration, at the midpoint between the lower border of the rib cage and the top of the iliac crest,^30^ and recorded nearest to the 0.1 cm. The waist:hip ratio (WHR) was calculated from the WC and HC.

BMI was calculated from the weight divided by the square of the height (kg/m^2^). According to BMI values, individuals were classified as normal (18.5–24.9 kg/m^2^), overweight (25.0–29.9 kg/m^2^) and obese (≥ 30.0 kg/m^2^).^31^

Socio-economic status was classified into quartiles according to average monthly household income;^27^ first quartile (low socioeconomic class), second quartile (middle class), third quartile (upper middle class), and fourth quartile (upper class). Education was classified into three levels based on the number of years of education: low (≤ four years of education), middle (five to 12 years of education), and high (≥ 13 years of education).^27^

Blood pressure and heart rate were measured in triplicate in the non-dominant arm after five minutes of resting in a seated position with the arm at the level of the heart. These parameters were measured using a validated, automated digital oscillometric sphygmomanometer (Omron 705CP, Tokyo, Japan). The readings were repeated at three-minute intervals. The mean of the last two readings was recorded.

The pulse pressure (PP) was computed as the difference between basal systolic blood pressure (SBP) and diastolic blood pressure (DBP). Mean blood pressure (MBP) was computed as the DBP + (PP/3). Hypertension was defined as SBP ≥ 140 mmHg, and/or DBP ≥ 90 mmHg, and/or the use of antihypertensive drugs.

Both the ATP III^4^ and JIS^7^ criteria were used to define the metabolic syndrome. The ATP III definition was based on the presence of three or more of the following components: WC > 102 cm (men), 88 cm (women); SBP ≥ 130 mmHg and/or DBP ≥ 85 mmHg and/or BP-lowering treatment; fasting triglyceride levels ≥ 150 mg/dl (1.70 mmol/l) or treatment for hypertriglyceridaemia; HDL-C < 40 mg/dl (1.04 mmol/l) (men), 50 mg/dl (1.30 mmol/l) (women), or treatment for dyslipidaemia; fasting glucose level ≥ 110 mg/dl or on antidiabetic medication.

The JIS definition was based on the presence of three or more of the following components: WC ≥ 94 cm (men), 80 cm (women); SBP ≥ 130 mmHg and/or DBP ≥ 85 mmHg and/or BP-lowering treatment; fasting triglyceride levels ≥ 150 mg/dl (1.70 mmol/l) or treatment for hypertriglyceridaemia; HDL-C < 40 mg/dl (1.04 mmol/l) (men), 50 mg/dl (1.30 mmol/l) (women) or treatment for dyslipidaemia; fasting glucose level ≥ 100 mg/dl (5.55 mmol/l) or on antidiabetic medication.

## Statistical analysis

Data were analysed using SPSS software, version 13.0 (SPSS Inc, Chicago, IL). Continuous variables are reported as mean ± standard deviation, and compared by gender using the independent-samples *t*-test. Categorical variables were expressed as proportions and compared using the chi-square test or Fisher’s exact test if appropriate. Prevalence of the metabolic syndrome was age-standardised by direct method using as reference the world population distribution as projected by the WHO for 2000 to 2025.^32^ Age-specific prevalence of the metabolic syndrome was estimated per age decades (< 30, 30–39, 40–49, 50–59 and ≥ 60 years).

ROC curve analysis was performed to determine the appropriate cut-off points of WC for identifying subjects with two or more components of the metabolic syndrome (except for WC), as defined by the JIS criteria. For the purpose of this analysis, we considered the presence or absence of the metabolic syndrome as an outcome variable and WC as a testing variable.

Optimal values of WC were obtained from the Youden index [maximum (sensitivity + specificity – 1)].^33^ Positive predictive values (PPV) and negative predictive values (NPV) were also presented. The kappa coefficient was used to assess the statistical agreement between the ATP III and JIS criteria for identifying individuals with the metabolic syndrome. A *p*-value < 0.05 was considered statistically significant.

## Results

A complete data set was collected for 615 subjects (52.2% women). Compared with women (Table 1), men had higher mean values for height, WHR, creatinine and uric acid levels (all *p* < 0.001), and PP (*p* = 0.007). Women had higher mean values for HDL-C, WC, HC, BMI (all *p* < 0.001), and heart rate (*p* = 0.003). Age, weight, SBP, DBP, MBP, and glucose, total cholesterol, LDL-C, VLDL-C, and triglyceride levels were similar in both sexes.

**Table 1:** Characteristics of the participants according to gender

*Characteristics*	*All*	*Men*	*Women*	p*-value*
Number (%)	615 (100)	294 (47.8)	321 (52.2)	0.392
Age (years)	44.5 ± 10.6	45.1 ± 11.1	44.0 ± 10.1	0.176
Weight (kg)	68.6 ± 15.3	68.0 ± 14.9	69.2 ± 15.7	0.349
Height (cm)	163.3 ± 7.9	167.4 ± 7.1	159.6 ± 6.6	< 0.001
WC (cm)	82.1 ± 13.3	80.1 ± 12.9	83.9 ± 13.5	< 0.001
HC (cm)	95.7 ± 11.3	91.5 ± 9.4	99.5 ± 11.4	< 0.001
WHR	0.86 ± 0.09	0.87 ± 0.08	0.84 ± 0.09	< 0.001
BMI (kg/m^2^)	25.7 ± 5.4	24.1 ± 4.3	27.1 ± 5.8	< 0.001
SBP (mmHg)	134.7 ± 24.9	136.5 ± 22.7	133.0 ± 26.6	0.087
DBP (mmHg)	82.6 ± 14	82.7 ± 14.2	82.5 ± 13.8	0.862
PP (mmHg)	52.1 ± 14.9	53.8 ± 13.2	50.5 ± 16.2	0.007
MBP (mmHg)	100.0 ± 16.9	100.6 ± 16.4	99.4 ± 17.5	0.351
Heart rate (bpm)	68 ± 10	67 ± 10	69 ± 10	0.003
Glucose (mg/dl)	94.0 ± 21	94.9 ± 20	93.2 ± 21.8	
(mmol/l)	(5.22 ± 1.17)	(5.27 ± 1.11)	(5.17 ± 1.21)	0.313
Creatinine (mg/dl)	1.1 ± 0.2	1.2 ± 0.2	1.0 ± 0.2	
(μmol/l)	(97.24 ± 17.68)	(106.08 ± 17.68)	(88.40 ± 17.68)	< 0.001
Uric acid (mg/dl)	5.4 ± 1.7	6.1 ± 1.7	4.8 ± 1.4	< 0.001
TC (mg/dl)	191.5 ± 38.9	189.5 ± 41.4	193.2 ± 36.5	
(mmol/l)	(4.96 ± 1.01)	(4.91 ± 1.07)	(5.0 ± 0.95)	0.239
HDL-C (mg/dl)	46.0 ± 10.9	44.1 ± 10.3	47.6 ± 11.2	
(mmol/l)	(1.19 ± 0.28)	(1.14 ± .027)	(1.23 ± 0.29)	< 0.001
LDL-C (mg/dl)	125.5 ± 40.1	125.0 ± 41.8	125.9 ± 38.7	
(mmol/l)	(3.25 ± 1.04)	(3.24 ± 1.08)	(3.26 ± 1.0)	0.796
VLDL-C (mg/dl)	20.0 ± 8.0	20.4 ± 8.3	19.7 ± 7.7	
(mmol/l)	(0.52 ± 0.21)	(0.53 ± 0.21)	(0.51 ± 0.20)	0.339
TGL (mg/dl)	100.2 ± 40.0	101.8 ± 41.7	98.7 ± 38.4	
(mmol/l)	(1.13 ± 0.45)	(1.15 ± 0.47)	(1.12 ± 0.43)	0.339

Values are means ± standard deviation. WC, waist circumference; HC, hip circumference; WHR, waist-to-hip ratio; BMI, body mass index; SBP, systolic blood pressure; DBP, diastolic blood pressure; PP, pulse pressure; MBP, mean blood pressure; TC, total cholesterol; HDL-C, high-density lipoprotein cholesterol; LDL-C, low-density lipoprotein cholesterol; VLDL-C, very low-density lipoprotein cholesterol; TGL, triglycerides.

Table 2 shows distribution of risk factors, socio-economic and educational characteristics of the study population. Current smoking was higher in men (*p* = 0.035), whereas prevalence of overweight, obesity and low HDL-C levels were higher in women (all *p* < 0.001). However, prevalence of hypertension, diabetes, hypercholesterolaemia, hypertriglyceridaemia and high LDL-C levels were similar in both sexes (Table 2).

**Table 2:** Risk factors, educational level and socio-economic class of the study population

*Characteristics*	*All*	*Men*	*Women*	p*-value*
Hypertension, *n* (%)	278 (45.2)	136 (46.3)	142 (44.2)	0.615
Current smokers, *n* (%)	39 (6.3)	25 (8.5)	14 (4.4)	0.035
Diabetes, *n* (%)	35 (5.7)	16 (5.4)	19 (5.9)	0.799
Overweight, *n* (%)	180 (29.3)	80 (27.2)	100 (31.2)	< 0.001
Obesity, *n* (%)	120 (19.5)	27 (9.2)	93 (29.0)	< 0.001
High TC, *n* (%)	68 (11.1)	31 (10.5)	37 (11.5)	0.698
High TGL, *n* (%)	77 (12.5)	37 (12.6)	40 (12.5)	0.963
High LDL-C, *n* (%)	121 (19.7)	61 (20.7)	60 (18.7)	0.522
Low HDL-C, *n* (%)	308 (50.1)	108 (36.7)	200 (62.3)	< 0.001
Education level				0.926
Low, *n* (%)	213 (34.6)	110 (37.4)	103 (32.1)	
Medium, *n* (%)	150 (24.4)	69 (23.5)	81 (25.2)	
High, *n* (%)	252 (41.0)	115 (39.1)	137 (42.7)	
Socio-economic class				0.392
Low, *n* (%)	154 (25.0)	81 (27.6)	73 (22.7)	
Middle, *n* (%)	156 (25.4)	77 (26.2)	79 (24.6)	
Upper middle, *n* (%)	152 (24.7)	66 (22.4)	86 (26.8)	
Upper, *n* (%)	153 (24.9)	70 (23.8)	83 (25.9)	

Values are number of subjects (*n*) and percentages (%).

The overall crude prevalence of the metabolic syndrome was 17.6% [age-standardised: 8.7%, 95% confidence interval (CI): 6.8–11.3] for the ATP III criteria and 27.8% (age-standardised: 14.1.0%, 95% CI: 11.6–17.1) for the JIS criteria. As expected, the crude prevalence was higher in women than in men, irrespective of the criteria used (Table 3). In both sexes, the prevalence of the metabolic syndrome increased with age, however, women showed a higher prevalence in all age groups from 30 years and older (Table 3). Regarding socio-economic class and educational level (Table 4), there was no significant relationship of these factors with the metabolic syndrome in both sexes.

**Table 3:** Crude and age-standardised prevalence of the metabolic syndrome in men and women according to ATP III and JIS criteria

*Age group (years)*	*n*	*ATP III*	*JIS*
Men (*n* = 294)			
< 30	40	2 (5.0)	3 (7.5)
30–39	52	2 (3.8)	4 (7.7)
40–49	89	8 (9.0)	15 (16.9)
50–59	90	10 (11.1)	23 (25.6)
≥ 60	23	3 (13.0)	5 (21.7)
Total crude	294	25 (8.5)	50 (17.0)
Age-standardised	–	4.8	9.0
Women (*n* = 321)			
< 30	32	0 (0.0)	1 (3.1)
30–39	71	8 (11.3)	13 (18.3)
40–49	125	43 (34.4)	62 (49.6)
50–59	79	27 (34.2)	37 (46.8)
≥ 60	14	5 (35.7)	8 (57.1)
Total crude	321	83 (25.9)	121 (37.7)
Age-standardised (%)	–	12.6	19.2
Overall (n = 615)			
Crude	615	108 (17.6)	171 (27.8)
Age-standardised (%)	–	8.7	14.1

Values are *n* (%). ATP III, National Cholesterol Education Program Third Adult Treatment Panel; JIS, Joint Interim Statement.

**Table 4:** Prevalence of the metabolic syndrome from JIS criteria in men and women according to socio-economic class and educational level

	*Number (%)*	p*-value*
*Men*		
Socio-economic class		0.083
Low	8 (9.9)	
Middle	13 (16.9)	
Upper middle	11 (16.7)	
Upper	18 (25.7)	
Education level		0.444
Low	15 (13.6)	
Medium	12 (17.4)	
High	23 (20.0)	
*Women*		
Socio-economic class		0.199
Low	29 (39.7)	
Middle	28 (35.4)	
Upper middle	26 (30.2)	
Upper	38 (45.8)	
Education level		0.294
Low	45 (43.7)	
Medium	27 (33.3)	
High	49 (35.8)	

Values are number of subjects (*n*) and percentages (%).

In individuals diagnosed with the metabolic syndrome from the JIS definition (*n* = 171), the most frequent components were elevated blood pressure: 52.5% (men 55.4% vs women 49.82%, *p* = 0.165), reduced HDL-C levels: 50.1% (men 36.7% vs women 62.3%, *p* < 0.001) and high WC: 39.8% (men 15.3% vs women 62.3%, *p* < 0.001). The less frequent components were elevated glucose levels: 23.4% (men 25.9% vs women 21.2%, *p* = 0.172) and raised triglyceride levels: 10.7% (men 12.6% vs women 9.0%, *p* = 0.155).

Although the prevalence of the metabolic syndrome diagnosed from the JIS criteria was higher than with the ATP III criteria, there was good agreement between the two classifications in the overall sample [kappa = 0.712, (*p* < 0.001; 95% CI: 0.648–0.777)], as well as in men [kappa = 0.624 (*p* < 0.001; 95% CI: 0.493–0.755)] and in women [kappa = 0.731 (*p* < 0.001; 95% CI: 0.654–0.809)].

**Fig. 1.**
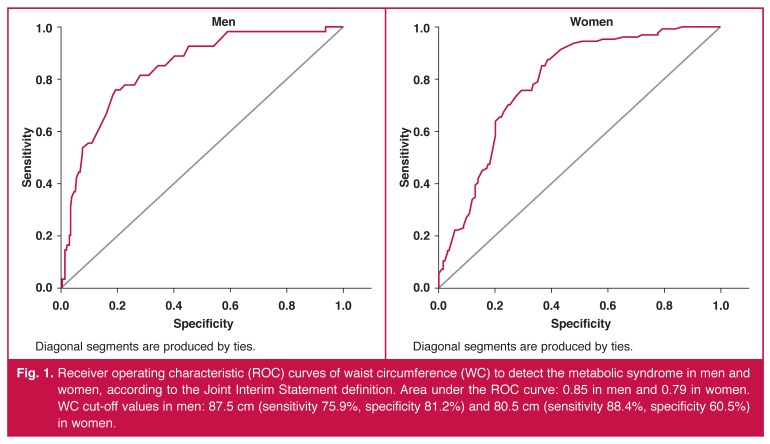
Receiver operating characteristic (ROC) curves of waist circumference (WC) to detect the metabolic syndrome in men and women, according to the Joint Interim Statement definition. Area under the ROC curve: 0.85 in men and 0.79 in women. WC cut-off values in men: 87.5 cm (sensitivity 75.9%, specificity 81.2%) and 80.5 cm (sensitivity 88.4%, specificity 60.5%) in women.

Fig. 1 shows results from the ROC curve analysis to identify subjects with two or more components of the metabolic syndrome using the JIS criteria. In men, the optimal cut-off value of WC to detect the metabolic syndrome with maximum sensitivity and specificity (Youden index = 0.563) was 87.5 cm (sensitivity 75.9%, 95% CI: 62.4–86.5; specificity 81.2%, 95% CI: 75.7–86; positive predictive value (PPV) 44.2%, 95% CI: 38.5–49.9 and negative predictive value (NPV) 94.2%, 95% CI: 91.5–96.9); whereas in women, the optimal cut-off value of WC (Youden index = 0.489) was 80.5 cm (sensitivity 88.4%, 95% CI: 81.3–93.5; specificity 60.5%, 95% CI: 53.4–67.3; PPV 57.5%, 95% CI: 52.1–62.9 and NPV 89.6%, 95% CI: 87.9–91.3).

There was good accuracy (*p* < 0.001) of the cut-off values of the WC to predict other components of the metabolic syndrome, as suggested by values of the area under the ROC curve [men: 0.85 (95% CI: 0.80–0.89) and women: 0.79 (95% CI: 0.74–0.84)].

## Discussion

The main findings of this study were a high prevalence of the metabolic syndrome among our subjects and a different cut-off value for WC for the diagnosis of the metabolic syndrome from those recommended for Africans by other studies.^5^,^7^ To our knowledge, this is the first study reporting the prevalence of the metabolic syndrome in Angolans.

Worldwide, the metabolic syndrome is increasingly becoming a pandemic,^7^ the level of prevalence being estimated to be 17–25% in the general population. However, estimates in sub-Saharan African populations are scarce and inaccurate.^11^ The crude prevalence in this study was in an intermediate point of the range (0–50%) reported for different African populations.^11^

The three most frequent components of the metabolic syndrome were elevated blood pressure, low HDL-C levels and elevated WC. A similar cluster of components was reported in an urban population in Kenya,^20^ and in a study including West Africans (Nigeria and Ghana) and African-Americans.^34^ Other studies reported a combination of high WC and low HDL-C levels as the most frequent components in Africans with high a prevalence of the metabolic syndrome.^14^,^18^,^25^

Although the underlying mechanisms are not fully understood, the increasing prevalence of the metabolic syndrome has been associated with a sedentary lifestyle and obesity.^7^ Also, it has been reported that in contrast to developed nations, in some African nations, a higher socio-economic status has been associated positively with increased obesity.^35^

In our study, distribution of the metabolic syndrome according to socio-economic class, defined by average household monthly income, was not significant. However, this study also showed a high prevalence of both obesity and overweight (47.8%) and hypertension (45.2%). The three most common components of the metabolic syndrome were elevated blood pressure, low HDL-C levels and high WC, suggesting a high risk for CV diseases in this occupational cohort. Therefore, considering the on-going socio-economic changes in Angola, the findings of this study may reflect the impact of the nutritional transition, behavioural and occupational changes, environmental risk factors and unhealthy lifestyle (mainly sedentary) with rapid weight gain, and the high consumption of salty and high caloric food.

Although this study showed a good concordance between the two criteria, the crude prevalence estimated with the JIS definition was 10.2% higher than that estimated with ATP III. This difference was mainly attributed to the different cut-off point for WC, which is lower for JIS than for ATP III criteria.

It is known that WC reflects both visceral and subcutaneous fat depots, but it has been used as a crude but relevant index of visceral adiposity. The role of visceral adiposity in the development of each metabolic syndrome component has been shown in non-African populations.^36^-^39^ In sub-Saharan African populations, a high WC was suggested as a key determinant for development of the metabolic syndrome.^14^

However, since country-specific cut-off values of WC still need to be defined for Africans, the cut-off values of WC derived from European population groups have been recommended for Africans.^5^,^7^ Emerging data suggested that African-specific cut-off values would be different from European cut-off values currently recommended by the IDF.^18^,^24^,^25^ In this study, the cut-off values for men were lower than that currently recommended for Africans (87.5 instead of 94 cm);^5^,^7^ whereas for women, these cut-off values were similar to those recommended for European and African women (80.5 vs 80 cm).

A few studies have attempted to establish cut-off values of WC for African groups,^18^,^24^,^25^ and they found different cut-off values from those currently recommended. In our study, the value of 87.5 cm for men is similar to that reported in South African studies of African men (86 cm),^18^ but different for women.^18^,^25^ However, our findings differed from those reported for men and women in another study of the same population (men: 90 cm, women: 98 cm).^24^

Discordant cut-off values of WC between different studies are to be expected since even in the same ethnic group, the WC may vary according to the country, as emphasised by the IDF5 and the JIS.^7^ Furthermore, it has been reported that variation in WC cut-off values obtained using the sensitivity and specificity approach were strongly correlated with mean levels of WC in the population.^40^,^41^ The cut-off values increased linearly with increasing population means, independent of WC measurement techniques and regardless of whether the health outcome was hypertension, dyslipidaemia, hyperglycaemia or a cluster of multiple outcomes.^40^ However, it remains to be clarified whether this variation was due to biological characteristics or the methodological approaches used to define the best cut-off point.^40^

In this study, women had higher mean values of WC than men (Table 1). It is known that the proportion of total fat in subcutaneous depots is higher in women than men.^42^ Therefore there is a potential risk of misclassification of women as having excessive visceral adiposity by using values of WC to predict other components of the metabolic syndrome. To minimise this difficulty in this study and ensure a correct classification for only women with strong evidence of two or more components of the metabolic syndrome, we selected the best cut-off values of WC, as suggested by the higher values of the Youden index. Therefore, this study reinforces the opinion that definition of cut-off values of WC should be country- and gender-specific.

There was a potential limitation to this study. Because we studied a convenient sample consisting of staff of a public university, our findings may not apply to the Angolan population as a whole. As previously detailed,^27^ however, participants were recruited from all higher education institutions, which represented university staff in the whole country. When this study was designed in 2009, all university staff were invited to take part. The study group included all occupational and socio-economic classes, including teachers and non-teaching workers.^26^,^27^

## Conclusion

There was a high prevalence of the metabolic syndrome in this occupational cohort, with a higher prevalence among women. This study suggested that optimal cut-off values of WC of 87.5 and 80.5 cm would be appropriate for the diagnosis of the metabolic syndrome in men and women, respectively. This may imply that the prevalence would have been different from that reported in this study if these values had been used. Further investigation is therefore needed to confirm optimal cut-off values of WC in the general Angolan population, in order to consistently estimate the trends of cardiometabolic risk factors in African populations.

## References

[1] Lakka HM, Laaksonen DE, Lakka TA, Niskanen LK, Kumpusalo E, Tuomilehto J (2002). The metabolic syndrome and total and cardiovascular mortality in middle-aged men.. J Am Med Assoc.

[2] Ford ES (2005). Risks for all-cause mortality, cardiovascular, and diabetes associated with the metabolic syndrome: a summary of the evidence.. Diabetes Care.

[3] Alberti KG MM, Zimmet PZ (1998). Definition, diagnosis and classification of diabetes mellitus and its complications. Part 1: diagnosis and classification of diabetes mellitus provisional report of a WHO consultation.. Diabet Med.

[4] (2001). Executive summary of the third report of the National Cholesterol Educational Program (NCEP) expert panel on detection, evaluation, and treatment of high blood cholesterol in adults (Adult Treatment Panel III).. J Am Med Assoc.

[5] Alberti KGMM, Zimmet P, Shaw J (2006). Metabolic syndrome: a new world-wide definition. A Consensus Statement from the International Diabetes Federation.. Diabet Med.

[6] Eckel RH, Grundy SM, Zimmet PZ (2005). The metabolic syndrome.. Lancet.

[7] Alberti KG MM, Eckel RH, Grundy SM, Zimmet PZ, Cleeman JI, Donato KA (2009). Harmonizing the metabolic syndrome: A joint interim statement of the International Diabetes Federation task force on epidemiology and prevention; National Heart, Lung, and Blood Institute; American Heart Association; World Heart Federation; International Atherosclerosis Society; and International Association for the Study of Obesity.. Circulation.

[8] Grundy SM (2008). Metabolic syndrome pandemic.. Arterioscler Thromb Vasc Biol.

[9] (2000). Non-Communicable Diseases: A Strategy for the African Region..

[10] Yusuf S, Reddy S, Ounpuu S, Anand S (2001). Global burden of cardiovascular diseases: part I: general considerations, the epidemiologic transition, risk factors, and impact of urbanization.. Circulation.

[11] Okafor IC (2012). The metabolic syndrome in Africa: Current trends.. Indian J Endocrinol Metab.

[12] Makuyana D, Gomo Z, Munyombwe T, Matenga JA, Hakim JG (2004). Metabolic syndrome disorders in urban black Zimbabweans with type 2 Diabetes mellitus.. Cent Afr J Med.

[13] Ntyintyane LM, Panz VR, Raal FJ, Gill GV (2006). Metabolic syndrome, undiagnosed diabetes mellitus and insulin resistance are highly prevalent in urbanised South African blacks with coronary artery disease.. Cardiovasc J S Afr.

[14] Fezeu L, Balkau B, Kengne AP, Sobngwi E, Mbanya J-C (2007). Metabolic syndrome in a sub-Saharan African setting: central obesity may be the key determinant.. Atherosclerosis.

[15] Ker JA, Rheeder P, Van Tonder R (2007). Frequency of the metabolic syndrome in screened South African corporate executives.. Cardiovasc J South Afr.

[16] Ntandou G, Delisle H, Agueh V, Fayomi B (2009). Abdominal obesity explains the positive rural-urban gradient in the prevalence of the metabolic syndrome in Benin, West Africa.. Nutr Res.

[17] Oladapo OO, Salako L, Sodiq O, Shoyinka K, Adedapo K, Falase AO (2010). A prevalence of cardiometabolic risk factors among a rural Yoruba south-western Nigerian population: a population-based survey.. Cardiovasc J Afr.

[18] Motala A, Esterhuizen T, Pirie FJ, Omar MAK (2011). The prevalence of metabolic syndrome and determination of the optimal waist circumference cut-off points in a rural South African Community.. Diabetes Care.

[19] Akpalu J, Akpalu A, Ofei F (2011). The metabolic syndrome among patients with cardiovascular disease in Accra, Ghana.. Ghan Med J.

[20] Kaduka LU, Kombe Y, Kenya E, Kuria E, Bore JK, Bukania ZN, Mwangi M (2012). Prevalence of metabolic syndrome among an urban population in Kenya.. Diabetes Care.

[21] Kengne AP, Limen SN, Sobngwi E, Djouogo CFT, Nouedoui C (2012). Metabolic syndrome in type 2 diabetes: comparative prevalence according to two sets of diagnostic criteria in sub-Saharan Africans.. Diabet Metab Synd.

[22] Balkau B, Charles MA (1999). Comment on the provisional report of WHO consultation. European Group for the Study of Insulin Resistance (EGIR).. Diabet Med.

[23] Lin WY, Lee LT, Chen CY, Lo H, Hsia HH, Liu IL, Lin RS, Shau WY, Huang KC (2002). Optimal cut-off values for obesity: using simple anthropometric indices to predict cardiovascular risk factors in Taiwan.. In J Obes Relat Metab Disord.

[24] Prinsloo J, Malan L, de Ridder JH, Potgieter JC, Steyn HS (2011). Determining the waist circumference cut-off which best predicts the metabolic syndrome components in urban Africans: The SABPA Study.. Exp Clin Endocrinol Diabetes.

[25] Crowther NJ, Norris SA (2012). The current waist circumference cut point used for the diagnostic of metabolic syndrome in Sub-Saharan African women is not appropriate.. PLOS One.

[26] Magalhães P, Capingana DP, Silva ABT, Ferreira AVL, Sá Cunha R, Rodrigues SL (2013). Age- and gender-specific reference values of pulse wave velocity for African adults: Preliminary results.. Age.

[27] Capingana DP, Magalhães P, Silva ABT, Gonçalves MAA, Baldo MP, Rodrigues SL, Simões CCF, Ferreira AVL, Mill JG (2013). Prevalence of cardiovascular risk factors and socioeconomic level among publicsector workers in Angola.. BMC Public Health.

[28] Friedewald WT, Levy RI, Fredrickson DS (1972). Estimation of the concentration of low-density lipoprotein cholesterol in plasma, without use of the preparative ultracentrifuge.. Clin Chem.

[29] Pereira AC, Sposito AC, Mota GF, Cunha RS, Herkenhoff FL, Mill JG (2006). Endothelial nitric oxide synthase gene variant modulates the relationship between serum cholesterol levels and blood pressure in the general population: new evidence for a direct effect of lipids in arterial blood pressure.. Atherosclerosis.

[30] Lean MEJ, Han TS, Morrison CE (1995). Waist circumference as a measure for indicating need for weight management.. Br Med J.

[31] (1998). Obesity: Preventing and managing the global epidemic: Report of a WHO Consultation on Obesity, Geneva, 3–5 June 1997. http://whqlibdoc.who.int/hq/1998/WHO_NUT_NCD_98.1_(p1-158).pdf.>.

[32] Ahmad OB, Boschi-Pinto C, Lopez AD, Murray CJL, Lozano R, Inoue M (2001). Age standardization of rates. A new WHO standard. EIP/GPE/EBD. GPE discussion paper Series.. WHO.

[33] Youden WJ (1950). An index for rating diagnostic tests.. Cancer.

[34] Sumner AE, Zhou J, Doumatey A, Imoisili OE, Amoah A, Acheampong J (2010). Low HDL-cholesterol with normal triglycerides levels is the most common lipid pattern in West Africans and African Americans with metabolic syndrome: Implications for cardiovascular disease prevention.. CVD Prev Control.

[35] Fezeu L, Mincoulou E, Balkau B, Kengne AP, Awah P, Unwin N (2006). Association between socioeconomic status and obesity in urban Cameroon.. Int J Epidemiol.

[36] Boyko EJ, Leonetti DL, Bergstrom RW, Newell-Morris L, Fujimoto WY (1996). Visceral adiposity, fasting plasma insulin, and lipid and lipoprotein levels in Japanese Americans.. Int J Obes Relat Metab Disord.

[37] Boyko EJ, Fujimoto WY, Leonetti DL, Newell-Morris  L (2000). Visceral adiposity and risk of type 2 diabetes: a prospective study among Japanese Americans.. Diabetes Care.

[38] Hayashi T, Boyko EJ, Leonetti DL, McNeely MJ, Newell-Morris L, Khan SE (2003). Visceral adiposity and the risk of impaired glucose tolerance: a prospective study among Japanese Americans.. Diabetes Care.

[39] Hayashi T, Boyko EJ, Leonetti  DL, McNeely MJ, Newell-Morris L, Khan SE (2004). Visceral adiposity is an independent predictor of incident hypertension in Japanese Americans.. Ann Intern Med.

[40] Wang Z, Ma J, Si D (2010). Optimal cut-off values and population means of waist circumference in different populations.. Nutr Res Rev.

[41] Takahara M, Katakami N, Kaneto H, Noguchi M, Shimomura I (2012). Statistical reassessment of the association between waist circumference and clustering metabolic abnormalities in Japanese population.. J Atheroscler Thromb.

[42] Hayashi T, Boyko EJ, Mc-Neely MJ, Leonetti DL, Khan SE, Fujimoto WY (2007). Minimum waist and visceral fat values for identifying Japanese Americans at risk for the metabolic syndrome.. Diabetes Care.

